# Efficacy of MRI Over Ultrasound in Evaluation of Abnormal Uterine Bleeding With Histopathological Correlation

**DOI:** 10.7759/cureus.38560

**Published:** 2023-05-04

**Authors:** Kalathuru Uhasai, Deepti Naik, Rathnamma P

**Affiliations:** 1 Department of Radiology, Sri Devaraj Urs Academy of Higher Education and Research, Kolar, IND; 2 Department of Obstetrics and Gynecology, Sri Devaraj Urs Academy of Higher Education and Research, Kolar, IND

**Keywords:** malignancy, leiomyoma, adenomyosis, polyp, histopathology, mri, usg, aub

## Abstract

Introduction

Abnormal uterine bleeding (AUB) is one of the most common problems in gynecologic practice. In the peri- and postmenopausal age group, it accounts for more than 70% of all gynecological complaints. The current study's objective was to compare the effectiveness of MRI and ultrasonography (USG) in detecting the cause of abnormal uterine bleeding with pathological correlation.

Material and methods

We conducted an observational study involving subjects with abnormal uterine bleeding. Patients who presented with abnormal uterine bleeding were referred to the department of radiodiagnosis and underwent USG of the abdomen and pelvis, followed by an MRI of the pelvis. Findings were analyzed and compared with the histopathological examination (HPE) of the samples of hysterectomized uterus, polypectomy, myomectomy, and dilation and curettage (D&C) of the endometrium.

Results

Among the study population, USG reports showed two patients (4.10%) with polyps, seven patients (14.58%) with adenomyosis, 25 patients (52.08%) with leiomyomas, and 14 patients (29.16%) with malignancies. On MRI examination, three patients (6.25%) were diagnosed with polyps, nine patients (18.7%) with adenomyosis, 22 patients (45.8%) with leiomyomas, and 14 patients (29.16%) were reported to have malignancies. The measure of agreement with the kappa value for MRI and HPE in evaluating the causes of abnormal uterine bleeding is 1.0 (very good). Whereas the kappa agreement value of USG and HPE in evaluating the causes of abnormal uterine bleeding is 0.903 (acceptable). The sensitivity of USG in diagnosing polyps, adenomyosis, leiomyoma, and malignancy was observed at 66%, 77.78%, 100%, and 100%, respectively. The sensitivity of MRI in diagnosing polyps, adenomyosis, leiomyoma, and malignancy was 100% for each.

Conclusion

MRI is the most effective method for accurate identification of the location, number, and characterization of lesions, extensions, and staging of carcinomas.

## Introduction

At least one-third of women worldwide are likely to experience abnormal uterine bleeding (AUB) at some point during their lifetime. Uterine bleeding without pregnancy is referred to as AUB and is abnormal in terms of regularity, volume, frequency, or duration [1]. AUB can be classified as structural or nonstructural. Adenomyosis, uterine polyps, leiomyoma, and malignancy are structural causes of AUB.

Nonstructural causes include coagulation disorders, primary endometrial disorders, molecular deficiencies, ovulatory dysfunction, intrauterine devices, iatrogenic etiologies-exogenous gonadal steroids, and other unclassified causes [1,2]. Nonstructural causes will not be further discussed in this study as they cannot be determined through imaging. On the other hand, imaging can be used to diagnose structural causes. In perimenopausal women, adenomyosis, leiomyoma, and endometrial polyps are the most common causes of AUB [3]. AUB is the most common symptom of endometrial cancer, which is one of the most serious conditions in postmenopausal women. Thus, evaluation of AUB caused by structural causes such as polyps or endometrial hyperplasia is necessary [3-5]. Additionally, imaging is used to identify and treat AUB in women of reproductive age groups and postmenopausal women [3,5-7].

MRI has the potential to be a promising and accurate imaging technique when the clinical diagnosis cannot be validated, and the ultrasonography (USG) is misleading, especially when the patient has normal findings and still exhibits symptoms [8]. Transvaginal ultrasonography (TVS) is still the primary screening method, and MRI helps in staging and determining the severity of the disease [9,10]. In the pre-procedure examination for uterine artery embolization (UAE), pelvic MRI is frequently used to create a baseline, pinpoint the location and size of uterine fibroids, and assess vascular structure [11].

The current investigation was conducted to assess different structural causes of AUB using USG and MRI examination. The pathological diagnosis will be correlated with the imaging findings. By determining the root cause of AUB, this study aids in avoiding invasive procedures like hysteroscopy, diagnostic laparoscopy, and hysterectomies.

## Materials and methods

We conducted a hospital-based observational study among non-pregnant patients above 18 years with AUB who underwent USG and MRI of the pelvis and histopathological examination over a period of 18 months (from January 2021 to June 2022). The study was conducted at RL Jalappa Hospital and Research Centre attached to Sri Devaraj Urs Academy of Higher Education and Research Center in Karnataka. The sample size was calculated as 43 using Open Epi software version 3.01 [12]. The study was approved by the Institutional Ethics Committee of Sri Devaraj Urs Medical College, Kolar, Karnataka, India (with the approval number SDUMC/KLR/IEC/601/2020-21). Informed written consent was obtained from all the study participants, and only those participants willing to sign the informed consent were included in the study. The risks and benefits of the study and the voluntary nature of participation were explained to the participants before obtaining consent. The confidentiality of the study participants was maintained. Data were entered into Microsoft Excel (Microsoft® Corp., Redmond, US) and Statistical Package for the Social Sciences (SPSS; IMB Inc., Armonk, US) version 20 and was used for statistical analysis.

USG of the pelvis in patients with abnormal uterine bleeding was done with the help of a Philips EPIQ 5 (Bothell Everett Hwy, US) ultrasound machine or GE Voluson E6 (Chicago, US) ultrasound machine. MRI of the pelvis was performed using a 1.5T, 18-Channel MR Scanner (Magnetom Avanto, Siemens, Germany). We have obtained axial, coronal, and sagittal T2-weighted MR images and axial, coronal, and sagittal T1-weighted MR images. Diffusion-weighted imaging (DWI) was obtained in the axial plane by single-shot spin-echo and echo-planar imaging. Results were analyzed and correlated with histo-pathological examination (HPE) findings obtained from a sample of hysterectomized uterus, polypectomy, myomectomy, or dilatation and curettage (D&C) of the endometrium.

## Results

A total of 48 subjects were included in the final analysis. The mean age of the study population was 46.88±9.78 years, ranging between 27 to 67 years. The premenopausal age group (less than 45 years) had 19 patients, the perimenopausal age group (45 to 55 years) had 16, and the postmenopausal age group (more than 55 years) had 13 patients (Table 1).

**Table 1:** Age distribution in the study population (n=48)

Age group	Age	Number of patients
Premenopausal	<45	19
Perimenopausal	45-55	16
Postmenopausal	>55	13

Among the study population, 25 participants presented with menorrhagia, 11 participants presented with metrorrhagia, and 12 participants with polymenorrhea. Out of all cases, 10 had dysmenorrhea as an additional symptom, especially in patients with adenomyosis (Table 2).

**Table 2:** Descriptive analysis of symptoms with which patients presented (n=48) Menorrhagia - menstrual periods with abnormally heavy or prolonged bleeding; metrorrhagia - abnormal bleeding between regular menstrual periods; polymenorrhea - frequent, short menstrual cycles; dysmenorrhea - pain associated with menstruation

Symptom	Frequency	Percentages
Menorrhagia	25	52.08%
Metrorrhagia	11	22.91%
Polymennorrhea	12	25.00%
Dysmenorrhea	10	20.83%

Transabdominal scan (TAS) with or without TVS reported two cases of polyps, seven of adenomyosis, 25 cases of leiomyomas, and 14 cases of malignancies (Table 3).

**Table 3:** Descriptive analysis of causes of AUB on ultrasound (n=48) AUB - abnormal uterine bleeding

Cause	Frequency	Percentages
Polyp	2	4.10%
Adenomyosis	7	14.58%
Leiomyoma	25	52.08%
Malignancy	14	29.16%

Among the study population, three (6.25%) cases were reported as polyps, nine (18.7%) as adenomyosis, 22 (45.8%) as leiomyomas, and 14 (29.16%) were reported as malignancies on MR imaging (Table 4).

**Table 4:** Descriptive analysis of causes of AUB on MRI (n=48) AUB - abnormal uterine bleeding

Cause	Frequency	Percentages
Polyp	3	6.25%
Adenomyosis	9	18.75%
Leiomyoma	22	45.83%
Malignancy	14	29.16%

Among the study population, three (6.25%) were reported as polyps, nine (18.7%) were reported as adenomyosis, 22 (45.8 %) were reported as leiomyomas, and 14 (29.16 %) were reported as malignancies on HPE (Table 5).

**Table 5:** Descriptive analysis of causes of AUB on HPE (n=48) AUB - abnormal uterine bleeding; HPE - histopathological examination

Cause	Frequency	Percentages
Polyp	3	6.25%
Adenomyosis	9	18.75%
Leiomyoma	22	45.83%
Malignancy	14	29.16%

When comparing USG and HPE in the evaluation of abnormal uterine bleeding causes, the measure of agreement with the kappa value is 0.903 (acceptable). This difference is because of three lesions that were misdiagnosed on USG and correctly diagnosed on MRI. Among them, one polyp was misdiagnosed as a submucosal fibroid, and two focal adenomyosis were misdiagnosed as intramural fibroids on USG. This is shown in Tables 6 and 7.

**Table 6:** Crosstable of USG and HPE USG - ultrasonography; HPE - histopathological examination

Count		HPE				Total
		Adenomyosis	Leiomyoma	Malignancy	Polyp	
USG	Adenomyosis	7	0	0	0	7
	Leiomyoma	2	22	0	1	25
	Malignancy	0	0	14	0	14
	Polyp	0	0	0	2	2
Total		9	22	14	3	48

**Table 7:** Measure of agreement (kappa value) of USG and HPE (valid cases n=48) USG - ultrasonography; HPE - histopathological examination

Symmetric measures		Value	Asymptotic standard error	Approximate T	p-value
Measure of agreement	Kappa	0.903	0.054	9.375	0.001

Comparing MRI and HPE of evaluations of the causes of abnormal uterine bleeding, a measure of agreement with the kappa value is 1.0 (very good). MRI was able to correctly diagnose all the lesions. This is shown in Tables 8 and 9.

**Table 8:** Crosstable of MRI and HPE HPE - histopathological examination

Count		HPE				Total
		Adenomyosis	Leiomyoma	Malignancy	Polyp	
MRI	Adenomyosis	9	0	0	0	9
	Leiomyoma	0	22	0	0	22
	Malignancy	0	0	14	0	14
	Polyp	0	0	0	3	3
Total		9	22	14	3	48

**Table 9:** Measure of agreement (kappa value) of MRI and HPE (valid cases n=48) HPE - histopathological examination

Symmetric measures		Value	Asymptotic standard error	Approximate T	p-value
Measure of agreement	Kappa	1	0.001	10.582	0.001

The sensitivity of USG in diagnosing polyps, adenomyosis, leiomyoma, and malignancy was 66%, 77.78%, 100%, and 100%, respectively. The sensitivity of MRI in diagnosing polyps, adenomyosis, leiomyoma, and malignancy was 100% for each. This is shown in Table 10. 

**Table 10:** Sensitivity of USG and MRI in evaluating various causes of AUB USG - ultrasonography; AUB - abnormal uterine bleeding

Causes of AUB	Sensitivity on USG	Sensitivity on MRI
Polyp	66%	100%
Adenomyosis	77.78%	100%
Leiomyoma	100%	100%
Malignancy	100%	100%

**Figure 1:**
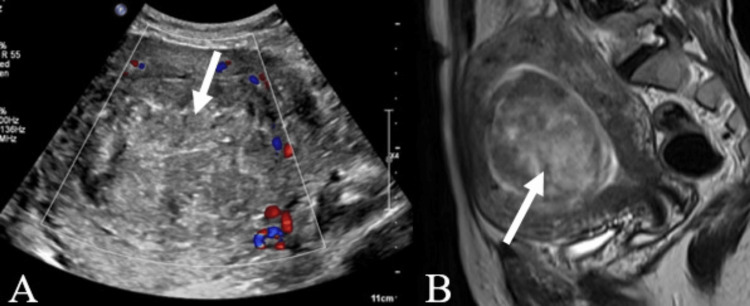
A case of a submucosal fibroid A: USG color Doppler image of the pelvis showing a heterogeneously hypoechoic lesion (white arrow) in the submucosal region of the uterus occupying the endometrial cavity showing peripheral vascularity. B: Sagittal T2-weighted pelvis MR image showing heterogeneously hyperintense lesion (white arrow) filling the endometrial cavity, consistent with submucosal leiomyoma. USG - ultrasonography

**Figure 2:**
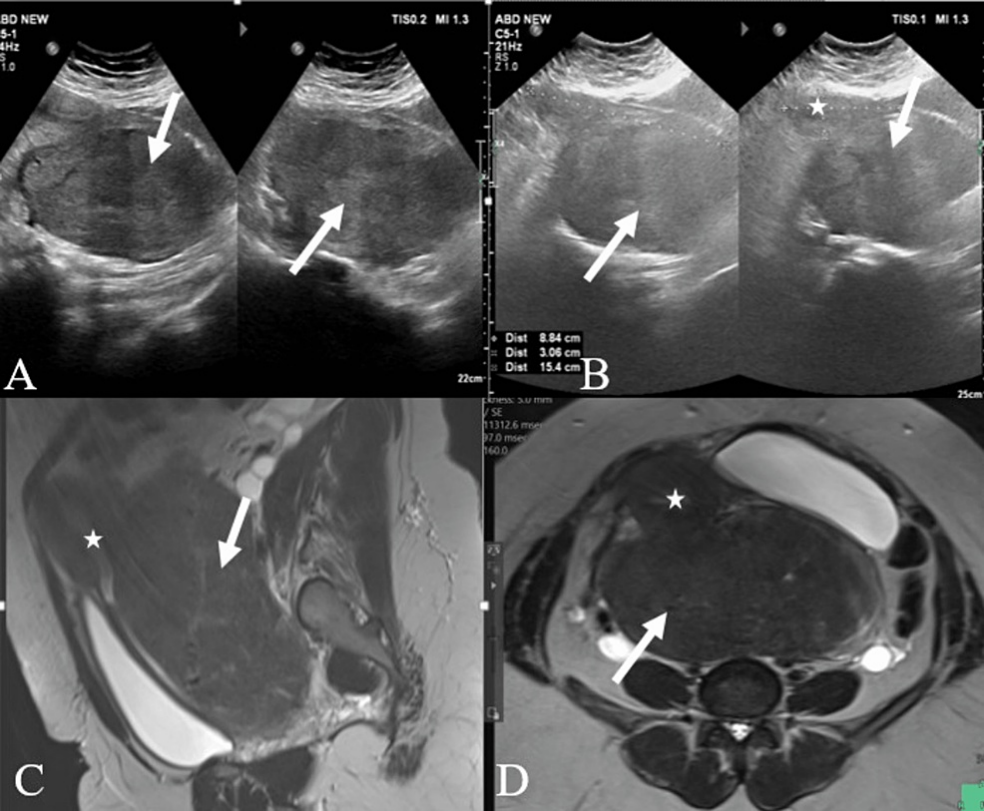
A case of large cervical fibroid A: USG pelvis transverse section showing a large heterogeneously iso-hypoechoic lesion in the pelvic cavity (white arrow). B: USG pelvis longitudinal section showing a large heterogeneously iso-hypoechoic lesion (white arrow) in the pelvic cavity pushing the uterus (star) anteriorly. The cervix is not visualized separately. C: Sagittal T2-weighted pelvis MR image showing heterogeneously hypointense lesion (white arrow) in pelvis arising from the cervix, consistent with cervical fibroid. D: Axial T2-weighted pelvis MR image showing heterogeneously hypointense lesion (white arrow) in pelvis arising from the cervix, pushing the uterus (star) anteriorly, consistent with cervical fibroid. USG -  ultrasonography

**Figure 3:**
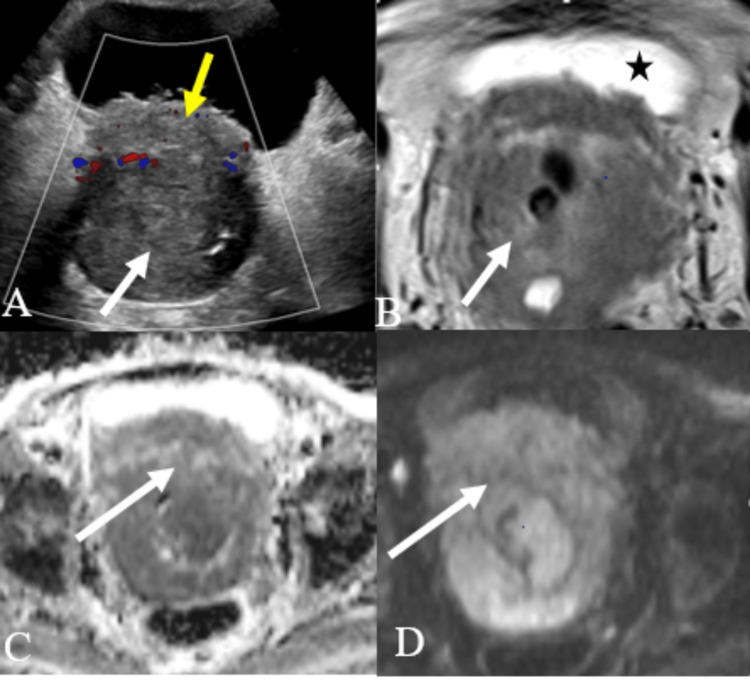
A case of cervical carcinoma A: USG color Doppler image of the pelvis showing bulky cervix with a heterogeneously hypoechoic lesion (white arrow), demonstrating internal vascularity. The lesion is seen infiltrating the posterior wall of the bladder (yellow arrow). B: Axial T2 pelvis MR image showing a large, fairly defined heterogeneously hyperintense lesion (white arrow) in the cervix. The lesion is seen infiltrating the posterior wall of the urinary bladder (black star). C and D: Axial section of DWI MRI of the pelvis and corresponding ADC where the lesion is showing patchy areas of restriction of diffusion (white arrow), suggestive of cervical malignancy. USG - ultrasonography; DWI - diffusion-weighted imaging; ADC - apparent diffusion coefficient

**Figure 4:**
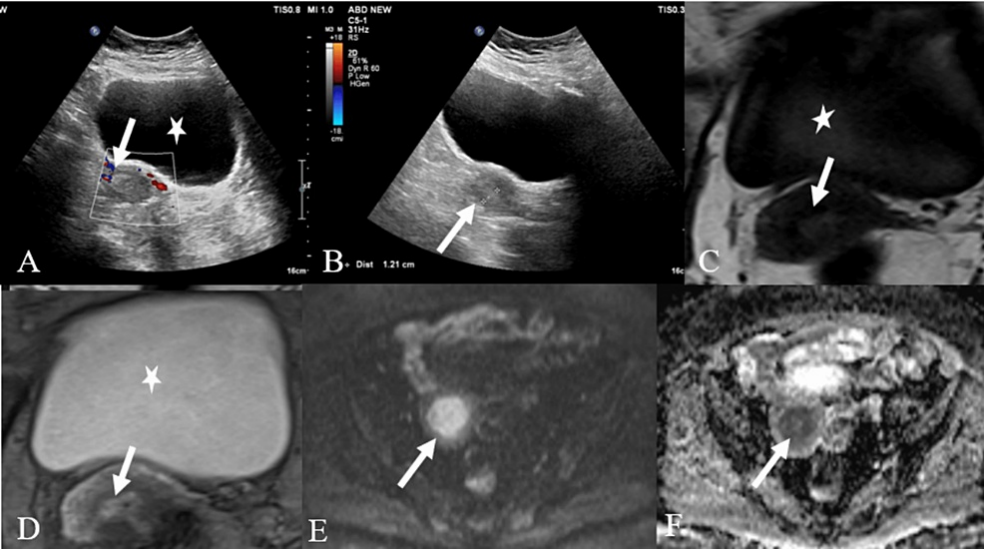
A case of endometrial carcinoma A: USG color Doppler image of the pelvis showing thickened and heterogenous endometrium (white arrow), demonstrating peripheral vascularity. B: USG of the pelvis showing thickened and heterogenous endometrium (white arrow). The uterus appears atrophied. The urinary bladder is denoted by a star. C and D: Sagittal T1 and T2 pelvis MR images showing thickened and heterogeneously hyperintense endometrium (white arrow). The urinary bladder is denoted by a star. E and F: Axial DWI of MRI of the pelvis and corresponding ADC images show the restriction of diffusion of thickened endometrium (white arrow), suggestive of endometrial malignancy. USG - ultrasonography; DWI - diffusion-weighted imaging; ADC - apparent diffusion coefficient

**Figure 5:**
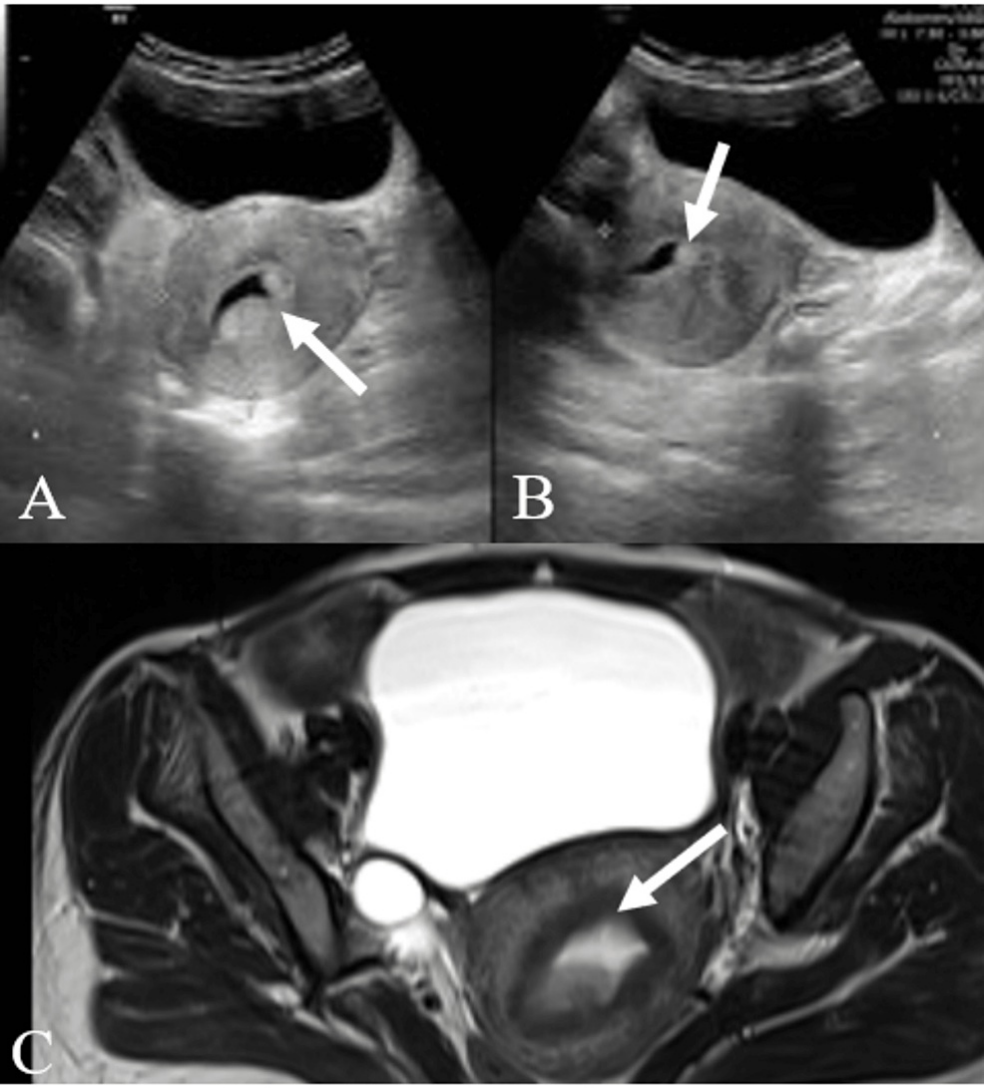
A case of endometrial polyp A and B: USG of the pelvis transverse and longitudinal sections showing two hyperechoic lesions (white arrow) in the endometrial cavity. C: MRI of the pelvis T2 weighted image showing three isointense lesions (white arrow) within the endometrial cavity. USG - ultrasonography

**Figure 6:**
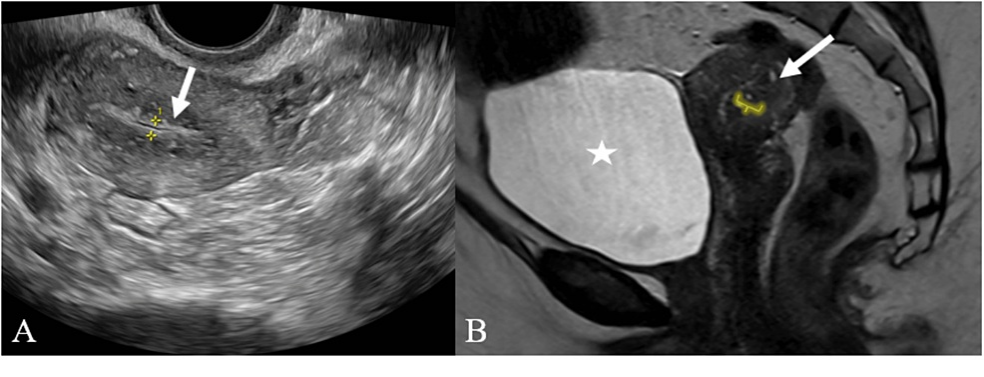
A case of adenomyosis A: USG of the pelvis longitudinal section showing sub-endometrial echogenic linear striations, extending from endometrium into inner myometrium (white arrow). Irregular endo-myometrial junction. Few hyperechoic islands were noted. B: Sagittal T2-weighted MRI of the pelvis showing widening of the junctional zone (yellow) and few cystic areas in anterior and posterior myometrium (white arrow). The urinary bladder is denoted by a star. USG - ultrasonography

In our study population, the total number of adenomyosis cases was nine, out of which seven cases were diffuse adenomyosis and two cases were focal adenomyosis, which were correctly diagnosed on MRI. Two cases were misdiagnosed as intramural fibroids on USG. The sensitivity of USG and MRI in detecting adenomyosis was 77.78% and 100%, respectively. Out of 48 cases, the total number of polyps was three cases, out of which USG was able to diagnose two cases with a sensitivity of 66.66%; one case was misdiagnosed as submucosal fibroid. All three cases were diagnosed as polyps on MRI with a sensitivity of 100%. Among the total of 48 cases, the sensitivity of USG and MRI in detecting fibroids and malignancies was the same, i.e., 100%, but MRI was superior in exactly identifying the location and number of lesions. MRI also helped in staging cases with malignancies. Study case images are shown in Figures 1-6.

## Discussion

Forty-eight patients referred from the Department of Obstetrics and Gynecology for evaluation of abnormal uterine bleeding underwent USG and MRI examination of the abdomen and pelvis. Patients underwent procedures like a cervical biopsy, endometrial curettage, myomectomy, or hysterectomy based on indication and followed by histopathological examination. The mean age of the study population was 46.88±9.78 years, ranging between 27 to 67 years. The premenopausal age group (less than 45 years) had 19 patients, the perimenopausal age group (45 to 55 years) had 16, and the postmenopausal age group (more than 55 years) had 13. The majority of the patients in our study belonged to the premenopausal age group. Among the study population, 25 (52.08%) participants presented with menorrhagia, 11 (22.9%) participants presented with metrorrhagia, and 12 (25%) participants presented with polymenorrhea. Out of all cases, 10 participants (20.83%) had dysmenorrhea (pain associated with menstruation) as an additional symptom, especially in patients with adenomyosis. The total number of patients was divided into four subcategories based on their histopathological diagnosis: polyp - three (6.25%), adenomyosis - nine (18.7%), leiomyoma - 22 (45.8%), and malignancies - 14 (29.16%). Ultrasound imaging reported two cases (4.10%) of polyps, seven (14.58%) cases of adenomyosis, 25 (52.08%) cases of leiomyomas, and 14 cases (29.16 %) were reported as malignancies. On MR imaging, three (6.25%) were reported as polyps, nine (18.7%) were reported as adenomyosis, 22 (45.8%) were reported as leiomyomas, and 14 (29.16%) were reported as malignancies. Sensitivity was calculated for all modalities in each subgroup and compared. While comparing MRI and HPE in the evaluation of abnormal uterine bleeding causes, the measure of agreement with the kappa value is 1.0 (very good). Whereas for USG and HPE evaluation of abnormal uterine bleeding causes, the measure of agreement with the kappa value is 0.903 (acceptable). This difference is because of three lesions that were misdiagnosed on USG and correctly diagnosed on MRI. One polyp was misdiagnosed as a submucosal fibroid, and two focal adenomyosis were misdiagnosed as intramural fibroids. MRI is the best tool for determining the location, number, and characterization of lesions; it provides the surgeon with preoperative mapping benefits. All infertile women undergoing uterine preservation surgery are required to undergo MRI before the procedure. Among patients with fibroids, cystic degeneration was seen in five patients on USG and seven patients on MRI. Four patients were showing calcifications on USG and two on MRI.

The present study cannot calculate specificity since we don't have true negative cases as compared with the normal population is not conducted. Out of 48 cases, there was a total of three polyp cases, out of which USG diagnosed two cases with a sensitivity of 66.66%; one case was misdiagnosed as submucosal fibroid. All three cases were diagnosed as polyps on MRI with a sensitivity of 100%. The total cases of adenomyosis were nine, out of which seven were diffuse adenomyosis and two were focal adenomyosis, which were correctly diagnosed on MRI. Two cases were misdiagnosed as intramural fibroids on USG. The sensitivity of USG and MRI in detecting adenomyosis was 77.78% and 100%, respectively. The sensitivity of USG and MRI in the detection of fibroids was the same, i.e., 100%, but MRI was superior in the exact identification of the location and number of lesions. There were 14 cases of malignancies in our total study population of 48. The sensitivity of USG and MRI in detecting malignancies was the same, which is 100%. However, MRI was better in the exact identification of the stage; further, MRI assessed the depth of myometrial/ parametrial involvement. Zawin et al. studied high-field MRI and USG in 23 women with leiomyomas [13]. In 21 of 23 ultrasound examinations, the endometrial stripe and junctional zone were not clearly visible. The researchers concluded that MRI detects more submucosal lesions than USG in women with leiomyoma. This is consistent with findings in our study, in which the exact origin of one large pelvo-abdominal fibroid was not apparent on TAS or TVS but was seen arising from the cervix on MRI. Thurnher concluded that if adenomyosis was diagnosed completely based on the thickness of the junctional zone (JZ) on images obtained on MRI examination, 5 mm must not be considered as a normal upper limit, as it might result in a greater number of false positives [14]. According to the standards established in a preceding study, the minimum JZ thickness in our study is 10 mm. Phillips et al. found that uterine biopsy or resectoscopic endometrial biopsy confirmed the MRI diagnosis of adenomyosis in all 20 patients [15]. Similarly, our study correctly identified all nine positive adenomyosis cases on MRI.

Ascher et al. conducted a study of over 20 patients on whom MRI, a transvaginal ultrasound, and HPE were performed [16]. Adenomyosis affected 17 people, and MRI diagnosed 15 of them correctly. Two false negative and one false positive diagnosis were found on MRI. Transvaginal ultrasound correctly diagnosed nine out of 17 cases. One false positive and eight false negatives were reported, with misinterpretation of adenomyosis as leiomyoma being the most common cause of false negative diagnosis [16]. Similarly, in our study, two fibroid cases diagnosed by USG turned out to be focal adenomyosis. In a research conducted by Mark et al. on 21 premenopausal patients, 12 of them were found to have fibroids, eight of them had adenomyosis, and one was normal [17]. On MRI, all eight adenomyosis were identified. MRI properly identified 10 out of 12 fibroids. In two cases it was challenging to distinguish between an adenomyosis and a fibroid, which is similar to our study. MRI is the preferred method for staging cervical cancer, preferably with contrast enhancement for a better ability to detect extensions. The surgical management is dramatically changed by the final MRI diagnosis. MRI provides a more accurate description of all uterine lesions. It allows for more accurate site localization and number detection and can reveal precise dimensions, the lesion's diameter, and degenerative changes. MR imaging provides a more accurate representation of the lesions' extent, which aids in staging the tumor.

Limitations

Few patients who met the inclusion criteria were unwilling to undergo an MRI as a diagnostic tool. The primary causes of this were the high cost and the general lack of understanding about how useful MRI is for early detection and diagnosis. There are no true negative cases, so the calculation of specificity was not possible.

## Conclusions

The measure of agreement with the kappa value for MRI and HPE in evaluating the causes of abnormal uterine bleeding is 1.0 (very good), and for USG and HPE, it is 0.903 (acceptable). MRI is the most effective method for detecting the location and number of lesions. The sensitivity of USG in diagnosing polyps was 66.66% since one case was misdiagnosed as submucosal fibroid, whereas the sensitivity of MRI in diagnosing polyps was 100%. The sensitivity of USG in diagnosing adenomyosis was 77.78%, and that of MRI in diagnosing adenomyosis was 100%.

The sensitivity of USG and MRI in detecting fibroids was the same, i.e., 100%; however, MRI was better at exactly identifying the location and the number of lesions. The sensitivity of USG and MRI in detecting malignancies was the same, i.e., 100%; however, MRI was better in assessing the exact staging and depth of myometrial/parametrial involvement. Ultimately, we conclude that MR imaging is a non-invasive, accurate, and well-tolerated technique for identifying the causes of abnormal uterine bleeding, with great histological correlation, when compared to USG. It is an excellent and accurate preoperative imaging modality for identifying, locating, and determining the size of lesions.
